# Dietary Different Replacement Levels of Fishmeal by Fish Silage Could Influence Growth of *Litopenaeus vannamei* by Regulating mTOR at Transcriptional Level

**DOI:** 10.3389/fphys.2020.00359

**Published:** 2020-05-06

**Authors:** Jianchun Shao, Lei Wang, Xuqing Shao, Mei Liu

**Affiliations:** ^1^Key Laboratory of Marine Biotechnology of Fujian Province, Institute of Oceanology, Fujian Agriculture and Forestry University, Fuzhou, China; ^2^Laboratory for Marine Biology and Biotechnology, Qingdao National Laboratory for Marine Science and Technology, Qingdao, China; ^3^CAS Key Laboratory of Experimental Marine Biology, Institute of Oceanology, Chinese Academy of Sciences, Qingdao, China; ^4^Shandong Cigna Detection Technology Co., Ltd., Qingdao, China

**Keywords:** *Litopenaeus vannamei*, fish silage, growth performance, intestinal histopathology, mammalian target of rapamycin signaling pathway

## Abstract

Fish silage (FS) has been confirmed as a high-quality feed ingredient because of its balanced nutrition, low cost, and environmental friendliness. In the present study, we evaluated the performance of replacing fishmeal by FS in the diet of white shrimp, *Litopenaeus vannamei*. Five isonitrogenous (410 g kg^–1^) and isolipidic (75 g kg^–1^) diets were formulated with replacement of fishmeal by 0% (FM), 25% (FS25%), 50% (FS50%), 75% (FS75%), and 100% (FS100%) FS. After an 8-week trial, shrimps fed low FS diets (FM and FS25%) had significantly higher final weight (FW), weight gain (WG), and specific growth ratio (SGR) (*P* < 0.05). No significant differences were found in body composition and most antioxidant enzyme activities of all groups (*P* > 0.05). Compared to high FS groups (FS75% and FS100%), low FS replacement levels (0 and 25%) had enhanced trypsin activity. And trypsin transcriptional level presented a similar trend with trypsin activity. In terms of intestinal histopathology, no obvious histological damage was observed in the intestine of all groups. *tor* and *s6k* of low replacement level groups (FM and FS25%) were significantly upregulated (*P* < 0.05), which indicated activation of mammalian target of rapamycin (mTOR) signaling pathway in low FS groups at transcriptional level. The enhanced performances of growth and mTOR signaling pathway in low FS groups (FM and FS25%) provided us some insights into the regulation mechanism of nutrient signal on growth. Based on the above, dietary FS could influence the growth of shrimp by regulating mTOR at the transcriptional level, and FS is a potential substitute of fishmeal in shrimp feed.

## Introduction

With the limitation of capture fishery production, aquaculture has been growing dramatically all over the world. Especially in 2014, aquaculture sector’s contribution to the food supply for humans overtook that of capture fishery for the first time (FAO, 2017). As the scale of aquaculture continues to expand, the demand for aquafeed has also increased. Fishmeal is recognized as the most important protein source in aquafeed industry because of its nutritional quality and palatability (Abasubong et al., 2018). However, due to climate change and overfishing, fishmeal shortages and its price surge. In addition, the excessive use of fishmeal may cause a series of environmental problems because of water and gas pollution during fishmeal production process (Sales, 2009). Therefore, finding alternative sources of fishmeal has become a hot spot for aquatic animal nutrition and feed research.

Farming of white shrimp, *Litopenaeus vannamei* is a fast-growing aquacultural activity in the world (Pauly and Froese, 2012). In 2016, the production of white shrimp around the world reached 4.16 million tons (FAO, 2018). Thus, the use of fishmeal in white shrimp feed increased enormously in the past, and finding alternative protein sources to replace fishmeal in shrimp feed is very essential. Recently, more and more potential protein sources, such as microalgae meal (Basri et al., 2014), fermented soybean meal (Shao et al., 2019), soy protein concentrate (Xie et al., 2016), protein hydrolysates (Shao et al., 2018), yeast extract (Zhao L. et al., 2017), and biofloc meal (Shao et al., 2017) were used to replace fishmeal in shrimp feed. Although some plant protein sources have achieved good results in fishmeal replacement research, the existence of anti-nutritional factors still constrained their application in actual production (Dossou et al., 2018). Hence, we pay much more attention to replacing fishmeal by animal protein sources.

Fish silage (FS) is produced from low-value fish or fish processing waste with liquification by adding a mixture of acids and enzymes (Haider et al., 2015). Due to its balanced nutrition, low cost, and environmental friendliness, FS is considered as a high-quality feed ingredient (Olsen and Toppe, 2017). And FS has been successfully used as a substitute of fishmeal in several fish species (Liang et al., 2006; Goosen et al., 2016). Recently, more and more studies were conducted to evaluate the effect of dietary FS in shrimp at different nutrient combination conditions (Gallardo et al., 2012; Rodríguez-González et al., 2018) and biofloc system (Gonçalves et al., 2019; Lobato et al., 2019). Here, we examined the performances of replacement of fishmeal in *L. vannamei* diet with FS. Additionally, many experiments have been carried out to evaluate the effect of fishmeal replacement. However, the underlying mechanism that limits fishmeal replacement remains largely unknown. Mammalian target of rapamycin (mTOR) signaling pathway is a regulator of cell growth, which could be modulated by stress, amino acids, and energy (Jewell et al., 2013). It regulates protein synthesis through ribosomal protein S6 kinase (s6k) and the eukaryotic translation initiation factor 4e-binding protein (4e-bp) (Wullschleger et al., 2006). This pathway has been described as an integration point which is closely related to nutrient sensing, metabolism, and growth in multiple species (Wullschleger et al., 2006; Loewith and Hall, 2011; Weisman, 2016). More and more evidence suggested the close relationship between mTOR and growth in aquatic animals (Tang et al., 2013; Tu et al., 2015; Liang et al., 2016). The study of mTOR signaling pathway in the present research provided us new insights into the nutrient sensing and growth in shrimp.

## Materials and Methods

### Feed Preparation

Fish silage powder was bought from Qingdao Blue Earthworm Corporation, and its composition was shown in Table 1. Five isonitrogenous (410 g kg^–1^) and isolipidic (75 g kg^–1^) diets were formulated with replacement of fishmeal by 0% (FM), 25% (FS25%), 50% (FS50%), 75% (FS75%), and 100% (FS100%) FS, respectively (Table 2). The essential amino acid profiles of diets were listed in Table 3, and the composition reached the requirements of shrimp. Feed production process was carried out as the previous description of our laboratory (Shao et al., 2018). Finished dry diets were stored at −20°C until use.

**TABLE 1:** Composition of the fish silage powder (g/100 g dry weight).

Items	Fish silage	Fishmeal
Crude protein	54.58	64.1
Crude lipid	3.54	9.5
Ash	12.05	14.2
Moisture	16.01	8.4
Protein hydrolysis	18.14	–
**Essential amino acid**		
Arginine	2.31	4.01
Histidine	1.81	2.37
Isoleucine	2.45	2.82
Leucine	3.84	4.66
Lysine	3.19	5.1
Methionine	1.66	2.75
Phenylalanine	2.01	2.64
Threonine	1.66	2.75
Valine	2.60	3.44

**TABLE 2:** Composition and proximate analysis of the experimental diets.

Ingredient (g/100 g)	FM	FS25%	FS50%	FS75%	FS100%
Fishmeal^a^	32	24	16	8	0
Fish silage^b^	0	10	20	30	40
Soybean meal^c^	35	35	35	35	35
Wheat flour^d^	22.2	19.7	17.3	14.9	12.5
Fish oil	2.8	3.3	3.7	4.1	4.5
Squid meal^e^	3	3	3	3	3
Soy lecithin	1	1	1	1	1
Brewer’s yeast	2	2	2	2	2
Vitamins premix^f^	1	1	1	1	1
Minerals premix^g^	1	1	1	1	1
**Proximate analysis**					
Moisture	9.17	9.52	9.19	9.58	9.43
Crude protein	41.14	40.87	41.59	41.36	40.79
Crude lipid	7.65	7.84	7.39	7.52	7.83
Ash	10.73	11.23	11.19	11.29	11.36
Gross energy (kJ g^–1^)^h^	16.84	16.73	16.75	16.68	16.71

*^*a*^Fishmeal: 64.1% crude protein, 9.5% crude lipid; Dale Feed Corporation, Yantai, China. ^*b*^Fish silage: 54.58% crude protein, 3.54% crude lipid; Qingdao Blue Earthworm Bio-Tech Corporation, Qingdao, China. ^*c*^Soybean meal: 42.8% crude protein, 1.2% crude lipid; Dale Feed Corporation, Yantai, China. ^*d*^Wheat flour: 15.0% crude protein, 0.8% crude lipid; Dale Feed Corporation, Yantai, China. ^*e*^Squid meal: 82.8% crude protein, 4.3% crude lipid; Dale Feed Corporation, Yantai, China. ^*f*^Vitamins premix (kg^–1^ of diet): vitamin A, 250,000 IU; riboflavin, 750 mg; pyridoxine-HCl, 400 mg; cyanocobalamin, 1 mg; thiamin, 250 mg; menadione, 250 mg; folic acid, 125 mg; biotin, 10 mg; a-tocopherol, 2.5 g; myo-inositol, 8,000 mg; calcium pantothenate, 1,250 mg; nicotinic acid, 2,000 mg; choline chloride, 8,000 mg; vitamin D3, 45,000 IU; vitamin C, 7,000 mg. ^*g*^Minerals premix (kg^–1^ of diet): ZnSO_4_ 7H_2_O, 0.04 g; CaCO_3_, 37.9 g; KCl, 5.3 g; KI, 0.04 g; NaCl, 2.6 g; CuSO_4_ 5H_2_O, 0.02 g; CoSO_4_ 7H_2_O, 0.02 g; FeSO_4_ 7H_2_O, 0.9 g; MnSO_4_ H_2_O, 0.03 g; MgSO_4_ 7H_2_O, 3.5 g; Ca (HPO_4_)_2_ 2H_2_O, 9.8 g. ^*h*^Gross energy was calculated using the factors as follows: protein, 23 kJ/g; lipid, 35 kJ/g; carbohydrates, 15 kJ/g (Molina-Poveda et al., 2013).*

**TABLE 3:** Essential amino acid profile (%) of experimental diets.

Amino acid	FM	FS25%	FS50%	FS75%	FS100%	Requirement for shrimps
Arginine	2.50	2.40	2.31	2.21	2.10	1.90^a^
Histidine	1.23	1.21	1.21	1.19	1.17	0.80^b^
Isoleucine	1.71	1.72	1.74	1.75	1.75	1.00^b^
Leucine	2.83	2.82	2.83	2.82	2.79	1.70^b^
Lysine	2.66	2.56	2.47	2.38	2.28	2.10^a^
Methionine	1.32	1.22	1.13	1.03	0.92	0.90^c^
Phenylalanine	1.76	1.74	1.73	1.70	1.66	1.40^b^
Threonine	1.64	1.58	1.53	1.46	1.41	1.40^d^
Valine	2.01	1.98	1.97	1.94	1.90	1.40^e^

*^*a*^Millamena et al. (1998). ^*b*^Millamena et al. (1999). ^*c*^Richard et al. (2010). ^*d*^Millamena et al. (1997). ^*e*^Teshima et al. (2002).*

### Feeding Trial

Pathogen-free shrimps used in this study were obtained from a commercial shrimp farm (Rizhao, Shandong, China). And the experiment was conducted in a test site of Institute of Oceanology in Qingdao City, China. Prior to the feeding trial, all shrimps were acclimated for 1 week in concrete ponds feeding with commercial feed (Dale Feed Corporation, Yantai, China). Then, 750 similar size shrimps (0.26 ± 0.03 g) were randomly distributed into 15 cylindrical tanks (500 L volume, containing 400 L water and 50 shrimps, 125 shrimps/m^3^) with water exchange and uninterrupted oxygenation system. The five experimental diets (FM, FS25%, FS50%, FS75%, and FS100%) were randomly assigned to 15 tanks with three replicates.

A total of 8-week feeding trial was performed. Feed was offered four times a day at 5:00, 11:00, 17:00, and 23:00. Daily ration was 3–5% of total body weight per tank. Residue was collected, and water exchange was conducted twice daily. During the experiment period, water quality was kept as follows: water temperature (28–31°C), pH (7.6–8.1), salinity (29–31 ppt), dissolved oxygen (5.6–6.2 mg/L), ammonia–nitrogen (0.12–0.20 mg/L), and nitrite–nitrogen (0.02–0.08 mg/L).

### Sample Collection

At the end of the feeding trial, all shrimps per tank were counted and weighed to calculate growth performance. Ten shrimps per tank were randomly collected and aseptically sacrificed. Hepatopancreas and muscle samples were rapidly frozen in liquid nitrogen and stored at −80°C for enzyme activity assays and real-time quantitative PCR analysis, and intestine was obtained for histopathology. Another 10 shrimps per tank were collected and stored at −20°C for analysis of body composition. All the experiments were conducted in accordance with the recommendations in the Guide for the Care and Use of Laboratory Animals of the National Institutes of Health (NIH). The study protocol and all experimental designs were conducted with approval from the Experimental Animal Ethics Committee of the Institute of Oceanology, Chinese Academy of Sciences.

### Chemical Analysis

Chemical analysis of experimental feed and shrimp body composition was performed according to standard methods (AOAC, 2012). Moisture was calculated following drying the samples at 105°C, and ash was determined by combustion in a muffle furnace at 550°C for 24 h. Crude protein was determined by nitrogen (N × 6.25) using the Kjeldahl method (Kjeltec TM8400, FOSS, Sweden). Crude lipid was measured by Soxhlet method (Buchi 36680, Switzerland).

### Enzyme Activity Assays

Hepatopancreas samples were homogenized and preprocessed as the previous study conducted by our laboratory (Shao et al., 2018). Digestive enzyme and antioxidant enzyme activities were analyzed with enzymatic kits according to the manufacturer’s protocol (Jiancheng, Nanjing, China). The source and information of each kit used in this study were as follows: lipase (Cat. No. A054-1-1), α-amylase (Cat. No. C016-1-1), trypsin (Cat. No. A080-1-1), superoxide dismutase (SOD; Cat. No. A001-3-2), glutathione peroxidase (GPX; Cat. No. A005-1-2), glutathione S-transferase (GST; Cat. No. A004-1-1), and catalase (CAT; Cat. No. A007-1-1).

## Histopathology

Intestine tissues of shrimp from FM, FS25%, FS50%, FS75%, and FS100% groups were fixed in 10% formalin for 24 h, dehydrated in an ascending alcohol series (50–95%). Dehydrated tissues were embedded in paraffin and sectioned into 4-μm thick with a microtome. The 4-μm-thick tissue sections were stained with hematoxylin and eosin (H&E), then examined using an ECHO microscope (California, CA, United States). To estimate histological damage degree, a scoring system was conducted as previous studies (Labrie et al., 2003; Abad-Rosales et al., 2010). Briefly, severe, moderate, mild, and none = 100%, <75%, <25%, and 0% of the fields with histological damage, compared to the control group (FM group).

### Gene Expression Analysis

Total RNA was extracted from hepatopancreas and muscle using RNA extraction kit according to the manufacturer’s instructions (Takara, Japan). The quality and yield of extracted total RNA were assessed by a 1.0% denaturing agarose gel and NanoDrop spectrophotometer (ND-2000, Thermo Fisher Scientific, United States). cDNA synthesis was performed using a One-Step gDNA Removal and cDNA Synthesis Kit according to the manufacturer’s recommendations (TransGen Biotech Co., Ltd., China). mTOR signaling pathway genes (*tor* and *s6k*) and *trypsin* were investigated in muscle and hepatopancreas, respectively. Specific primers were designed based on partial cDNA sequences in *L. vannamei* transcriptome analysis (Zhao W. et al., 2017), and β*-actin* was selected as a reference gene (Table 4). Real-time PCR was carried out in a sequence detection system (ABI7500, Thermo Fisher Scientific, United States) with three replicates of each sample as follows: 94°C for 30 s, then 40 temperature cycles of 94°C for 5 s and 60°C for 30 s. Gene expression level was analyzed using the 2^–ΔΔ*Ct*^ method (Livak and Schmittgen, 2001).

**TABLE 4:** Primers used for real-time quantitative PCR.

Gene name	Primer sequence (5′–3′)	Product size (bp)	Tm (°C)	PCR efficiency (%)
*tor*	F-TGCCAACGGGTGGTAGA	181	58	97
	R-GGGTGTTTGTGGACGGA			
*s6k*	F-GCAAGAGGAAGACGCCATA	210	59	97
	R-CCGCCCTTGCCCAAAACCT			
β*-actin*	F-GCCCATCTACGAGGGATA	121	57	99
	R-GGTGGTCGTGAAGGTGTAA			
*trypsin*	F-CGGAGAGCTGCCTTACCAG	141	59	98
	R-TCGGGGTTGTTCATGTCCTC			

### Calculations and Statistical Analysis

The parameters were calculated as follows:

$$\begin{array}{c} \text{Weight}\;\text{gain}\;(\text{WG},\;\%)\;\text{=}\;\text{100}\times ({\text{W}}_{\text{T}}{\text{–W}}_{\text{0}}){\text{/W}}_{\text{0}}\\ \text{Specific}\;\text{growth}\;\text{ratio}\;(\text{SGR},\;\%{\text{day}}^{\text{–}}{}^{\text{1}})\\ \text{= 100}\times (\text{Ln}\;{\text{W}}_{\text{T}}\text{–Ln}\;{\text{W}}_{\text{0}})\text{/T}\\ \text{Protein}\;\text{efficiency}\;\text{ratio}\;(\text{PER})\;\text{=}\;({\text{W}}_{\text{T}}{\text{–W}}_{\text{0}})\text{/protein}\;\text{intake}\\ \text{Feed}\;\text{efficiency}\;(\text{FE})\text{=}({\text{W}}_{\text{T}}{\text{–W}}_{\text{0}})\text{/feed}\;\text{consumed} \end{array}$$

W_*T*_ and W_0_ are final and initial weights (g), and T is the feeding period (56 days).

Statistical analysis was conducted using SPSS 19.0 (SPSS, Chicago, IL, United States). One-way ANOVA was used to test the main effect of dietary different feed on shrimps. Data throughout the text were presented as the means ± SD. Differences were compared using Duncan’s multiple range test after homogeneity of variance was checked. When *P-*value < 0.05, differences were considered statistically significant.

## Results

### Growth Performance

During the experimental period, shrimps fed with diets FM and FS25% performed significantly better in final weight (FW), WG, and SGR (*P* < 0.05), whereas diet FS100% significantly reduced growth performance of shrimps compared to the other dietary treatments (*P* < 0.05; Table 5). FE and PER decreased with increasing FS intake, which may be due to the poor nutritional properties of FS compared to fishmeal.

**TABLE 5:** Growth performance of shrimps fed with different experimental diets for eight weeks (means of triplicate ± SD).

	FM	FS25%	FS50%	FS75%	FS100%
FW (g)	5.07 ± 0.17^a^	4.95 ± 0.12^a^	4.35 ± 0.13^b^	3.61 ± 0.13^c^	2.65 ± 0.14^d^
WG (%)	1778 ± 62^a^	1734 ± 46^a^	1512 ± 48^b^	1238 ± 49^c^	881 ± 54^d^
SGR (%/day)	5.60 ± 0.06^a^	5.56 ± 0.04^a^	5.33 ± 0.05^b^	4.99 ± 0.06^c^	4.44 ± 0.09^d^
PER	1.83 ± 0.08^a^	1.73 ± 0.02^a^	1.55 ± 0.04^bc^	1.68 ± 0.09^ab^	1.51 ± 0.04^c^
FE	0.77 ± 0.03^b^	0.73 ± 0.01^b^	0.65 ± 0.02^b^	0.71 ± 0.04^b^	0.64 ± 0.06^a^

*FW, final weight; WG, weight gain; SGR, specific growth ratio; PER, protein efficiency ratio; FE, feed efficiency. Means in the same row not sharing a superscript letters are significantly different (*P* < 0.05).*

### Body Composition

Results of body composition were presented in Table 6. There were no significant differences in moisture, crude protein, crude lipid, and ash contents among five treatment groups (*P* > 0.05).

**TABLE 6:** Proximate moisture, protein, lipid, and ash (% of wet weight) composition of shrimps fed with different experimental diets for eight weeks (means of triplicate ± SD).

	FM	FS25%	FS50%	FS75%	FS100%
Moisture (%)	76.35 ± 0.29	76.57 ± 0.37	75.85 ± 0.22	76.45 ± 0.28	76.53 ± 0.38
Crude protein (%)	22.31 ± 0.55	22.64 ± 0.26	21.91 ± 0.23	21.79 ± 0.15	22.07 ± 0.31
Crude lipid (%)	0.76 ± 0.06	0.77 ± 0.04	0.79 ± 0.04	0.79 ± 0.05	0.81 ± 0.06
Ash (%)	1.51 ± 0.06	1.47 ± 0.04	1.53 ± 0.02	1.55 ± 0.03	1.49 ± 0.04

### Enzyme Activity

**FIGURE 1:**
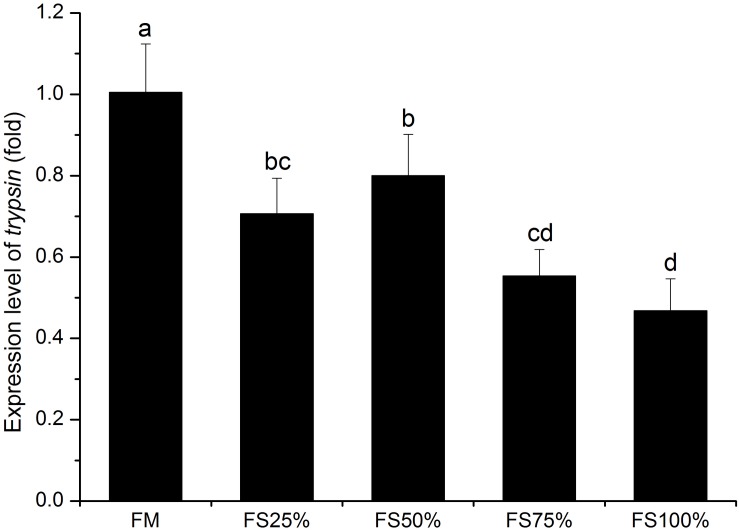
Relative expression level of *trypsin* in hepatopancreas of white shrimp (*Litopenaeus vannamei*) fed with five different diets. Results are shown as the mean ± SD, and different letters above a bar represent a significant difference (*P* < 0.05).

**FIGURE 2:**
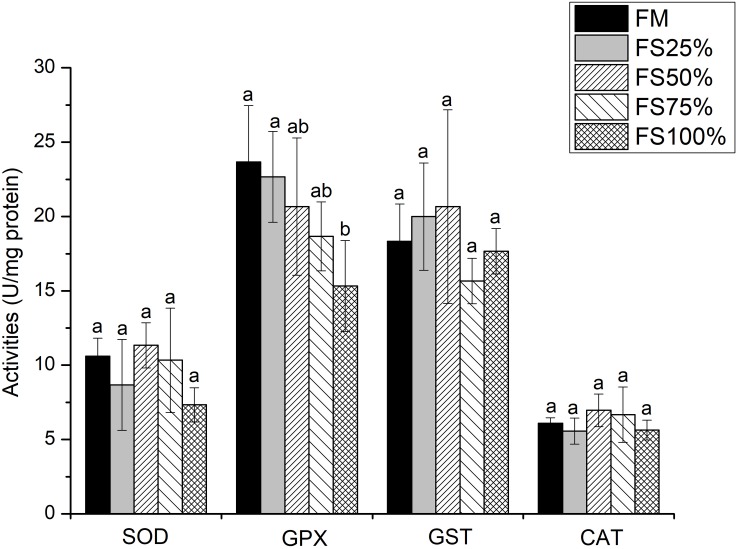
Specific activities of hepatopancreas antioxidant enzymes of shrimp fed with five different diets. Results are shown as the mean ± SD, and different letters above a bar represent a significant difference (*P* < 0.05). SOD, superoxide dismutase; GST, glutathione S-transferase; GPX, glutathione peroxidase; CAT, catalase.

No significant differences were observed in lipase and α-amylase activities among all dietary treatments (*P* > 0.05), whereas trypsin activity of FM and FS25% was significantly higher than that of FS75% and FS100% (*P* < 0.05; Table 7), which indicated that excessive FS intake negatively affected digestive enzyme activities. The mRNA transcript of *trypsin* in FM was significantly upregulated than that of diets with FS (*P* < 0.05; Figure 1). In terms of antioxidant enzymes, SOD, GST, and CAT enzyme activities showed no significant differences in all groups (*P* > 0.05), while GPX enzyme activity of FS100% was significantly lower than that of FM and FS25% (*P* < 0.05; Figure 2).

**TABLE 7:** Digestive enzyme activities in hepatopancreas of shrimps fed with different experimental diets for eight weeks (means of triplicate ± SD).

	FM	FS25%	FS50%	FS75%	FS100%
α-Amylase (U mg protein^–1^)	1.38 ± 0.16	1.51 ± 0.19	1.55 ± 0.13	1.52 ± 0.09	1.61 ± 0.04
Lipase (U mg protein^–1^)	0.80 ± 0.05	0.87 ± 0.13	0.89 ± 0.03	0.91 ± 0.07	0.82 ± 0.09
Trypsin (U mg protein^–1^)	115.51 ± 13.61^a^	109.79 ± 6.62^a^	105.52 ± 6.98^ab^	84.85 ± 7.53^c^	93.85 ± 3.32^bc^

*Means in the same row not sharing a superscript letters are significantly different (*P* < 0.05).*

### Intestinal Histopathology

**FIGURE 3:**
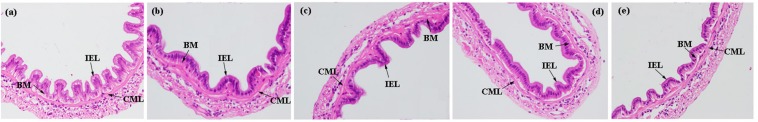
Histological analysis of intestine from of shrimp fed diets with 0 **(a)**, 25% **(b)**, 50% **(c)**, 75% **(d)**, and 100% **(e)** replacement of fishmeal by fish silage (FS). BM, basement membrane; CMLs, circular muscle layers; IEL, intestinal epithelial layer. Stained with hematoxylin and eosin (H&E), 200×.

Histological damage scores were presented in Supplementary Table S1. No obvious histological damage was observed in terms of basement membrane (BM) thickening, increased leukocyte infiltration, epithelial necrosis, and blood capillary hyperemia in the intestine of all groups (Figure 3). But compared to high replacement level groups (FS75% and FS100%), the BM and circular muscle layers (CMLs) of low replacement level groups (FM and FS25%) were more closely combined (Figure 3 and Supplementary Table S1).

### Mammalian Target of Rapamycin Signaling Pathway

**FIGURE 4:**
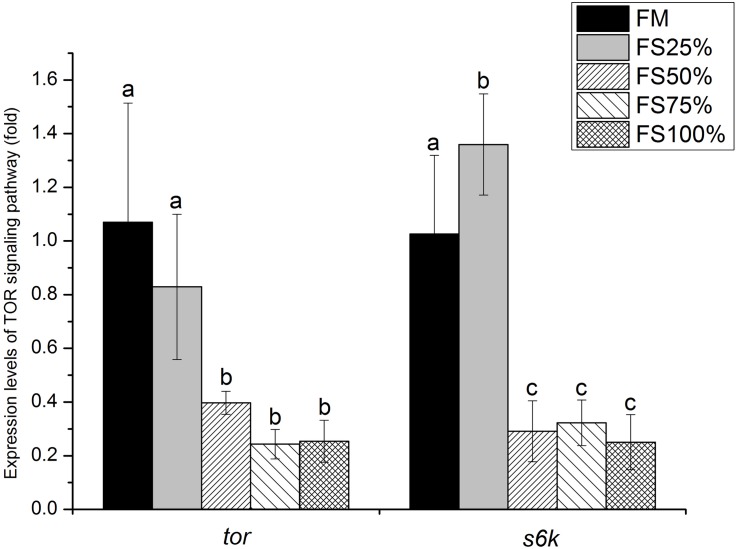
Relative expression levels of *tor* and *s6k* in muscle of white shrimp (*Litopenaeus vannamei*) fed with five different diets. Results are shown as the mean ± SD, and different letters above a bar represent a significant difference (*P* < 0.05).

Relative expression levels of mTOR signaling pathway genes in muscle were shown in Figure 4. *tor* expression level showed no significant difference between FM and FS25%. Both *tor* and *s6k* expression levels of FM and FS25% were significantly upregulated than those of FS50%, FS75%, and FS100% (*P* < 0.05).

## Discussion

Fish silage is a high-quality ingredient of feed, which has been successfully used in livestock and poultry, including quails (Panda et al., 2017), lambs (Tejeda-Arroyo et al., 2015), and pigs (Nørgaard et al., 2015). In the field of aquafeed, the use of FS on replacement of fishmeal also made significant progress. Based on the study of Liang et al. (2006), enhanced growth performance was obtained in Japanese sea bass (*Lateolabrax japonicus*) when 15% of fishmeal in a fishmeal-based diet was replaced by FS. And recently, the study on Mozambique tilapia (*Oreochromis mossambicus*) also documented that low replacement level of fishmeal with FS did not cause a negative effect on growth performance (Goosen et al., 2016). Many researchers also focused on the effects of dietary FS in shrimp. Rodríguez-González et al. (2018) found that a combination of 18.75% FS/39.75% *Jatropha curcas* kernel meals in shrimp diets made better growth performance. And in a biofloc system, up to 6% of tilapia silage inclusion did not compromise shrimp meat quality (Gonçalves et al., 2019). We evaluated growth performance and growth-regulating gene expression levels of replacement of fishmeal in *L. vannamei* diet with FS in the present study.

In this research, 25% replacement of fishmeal with FS had no effects on the growth performance of shrimps compared to FM group, while high replacement levels (75 and 100%) obviously had depressed performance in FW, WG, and SGR. Although FS is a high-quality protein source, its nutritional properties still have some deficiencies compared to fishmeal, such as crude protein and essential amino acid composition (Table 1). Excessive intake of FS in the diet can cause aquaculture animals to lose growth efficiency. Therefore, based on the result of growth performance, high replacement levels of fishmeal by FS is not a suitable option. Different from previous studies (Liang et al., 2006; Goosen et al., 2016), we designed a wide range of substitution (from 25 to 100%) of fishmeal with FS in our study. Previous studies conducted in fish did not use FS to replace more than 25% fishmeal. The effect of high replacement levels of fishmeal by FS on aquatic animals remains unknown. Therefore, we try to replace fishmeal with much more FS in shrimp feed. Although high replacement levels of fishmeal by FS did not achieve satisfactory results on growth performance, it is still a significative try to alleviate fishmeal shortage.

Overproduction of reactive oxygen species (ROS) and residual ROS cause serious damage to cells and tissues. To protect themselves against damage by ROS, cells have developed a set of antioxidant defense systems, involving many antioxidant enzymes, such as SOD, GST, GPX, and CAT (Duan et al., 2015). Therefore, antioxidative capacity is an important index to reflect the healthy status of crustaceans. In the present study, SOD, GST, and CAT enzyme activities showed no significant differences in all groups, and only GPX enzyme activity of FS100% decreased significantly. While many previous studies showed that dietary high levels of plant protein sources decreased more antioxidant enzyme activities in shrimp (Xie et al., 2016; Wan et al., 2018). FS is a better protein source without anti-nutritional factors than plant protein sources. The excellent nutritional properties of FS reduce its negative effect on the antioxidant enzyme activities of shrimp.

Intestinal histopathology was conducted to evaluate the intestinal health of shrimp fed with different levels of FS. No obvious histological damage was observed in the intestine of all groups, which indicated the excellent nutrition characteristics of FS for shrimp. It is also found that the BM and CMLs of low replacement level group (FM and FS25%) were more closely combined. The loose connection between BM and CMLs in high replacement level groups (FS75% and FS100%) may lead to barriers to nutrient absorption and transportation, which is related to the worse growth performance of high replacement level groups.

Trypsin plays a vital role in the assimilation of nutrition in hepatopancreas of shrimps (Muhliaalmazan, 2003). In our present study, dietary low FS replacement levels (0 and 25%) made positive effects on both trypsin activity and *trypsin* gene expression, which may contribute to growth of shrimp in low FS groups. Many previous studies also have found a close relationship between digestive enzyme activity and growth in shrimp. Based on the study of Pakravan et al. (2017), dietary 50% replacement level of fishmeal by microalgae stimulated trypsin activity and further enhanced growth of shrimp. More recently, Huang et al. (2019) found that dietary T-2 toxin decreased both digestive enzyme activity and growth performance. These evidences demonstrated the important effect of trypsin on grow performance in shrimp.

The vital role of trypsin in growth of shrimp has been verified by our present research and some previous studies (Sha et al., 2016; Pakravan et al., 2017), while the downstream regulation pathway of trypsin remains unknown. Amino acid concentration could be sensed to modulate mTOR signaling pathway activity and further regulate cell growth (Jewell et al., 2013). Trypsin activity directly affects the efficiency of protein digestion. In the present study, key genes (*tor* and s*6k*) of mTOR signaling pathway in low FS groups (FM and FS25%) were significantly upregulated at the transcriptional level. Interestingly, low FS groups also had higher trypsin activity and better growth performance. We speculated that trypsin activity influenced amino acid concentration in shrimp tissue and further provided mTOR signaling pathway with a signal to regulate cell growth. In addition, mTOR signaling pathway has been confirmed as an effector of cell growth and proliferation *via* the regulation of protein synthesis in model species and mammals (Miron et al., 2003; Fingar and Blenis, 2004; Loewith and Hall, 2011; Kakanj et al., 2016). In aquatic animals, several studies showed that dietary essential amino acid supplementation can activate TOR signaling pathway and further influenced the growth of fish (Tang et al., 2013; Ren et al., 2015; Wu et al., 2017). And Shao et al. (2018) found that dietary biofloc meal could make a difference on the key genes of mTOR signaling pathway and growth performance of shrimp. Therefore, we believe that different dietary replacement levels of fishmeal by FS could influence the growth of white shrimp by regulating the mTOR signaling pathway.

## Conclusion

Fish silage is a kind of high-quality feed ingredient because of its balanced nutrition, low cost, and environmental friendliness. In the present study, we evaluated the potential of replacing fishmeal by FS in feed of white shrimp. The results indicated that replacement of fishmeal by FS at 25% performed better than high replacement levels on growth performance. And we further found that dietary FS could regulate growth of shrimp by mTOR signaling pathway. In brief, FS is a potential substitute of fishmeal in shrimp feed to reduce the pressure of demand for fishmeal.

## Data Availability Statement

The datasets generated for this study are available on request to the corresponding author.

## Ethics Statement

The animal study was reviewed and approved by the Experimental Animal Ethics Committee of Institute of Oceanology, Chinese Academy of Sciences.

## Author Contributions

JS and ML made most contributions to this research, such as experiment design, sample collection, analysis of data, and drafting the manuscript, etc. LW made contribution to the design of this study and drafting the manuscript. XS participated in sample collection and analysis.

## Conflict of Interest

XS was employed by the company Shandong Cigna Detection Technology Co., Ltd. The remaining authors declare that the research was conducted in the absence of any commercial or financial relationships that could be construed as a potential conflict of interest.
