# Supplementary material for: Dietary Different Replacement Levels of Fishmeal by Fish Silage Could Influence Growth of Litopenaeus vannamei by Regulating mTOR at Transcriptional Level
Source: Front Physiol. 2020 May 6;11:359. doi: 10.3389/fphys.2020.00359 (PMC7232572; doi:10.3389/fphys.2020.00359)
Supplement: Supplementary file 1 [file Table_1.DOCX]

TABLE S1 Histological damage scores in intestine tissue of shrimps fed with different experimental diets for 8 weeks.

|  | FS25% | FS50% | FS75% | FS100% |
| --- | --- | --- | --- | --- |
| Basement membrane thickening | - | - | - | - |
| Increased leukocyte infiltration | - | - | - | - |
| Epithelial necrosis | - | - | - | - |
| Blood capillary hyperemia | - | - | - | - |
| Epithelial separation from basement membrane | - | × | ×× | ×× |

Severe (×××), moderate (××), mild (×), no damage (-) = 100%, < 75%, < 25 % and 0% of the fields with histological damage, compared to the control.
